# The Saturday Fund and Hospital Almoners

**Published:** 1913-05-24

**Authors:** 


### THE SATURDAY FUND AND HOSPITAL ALMONERS.

In The Hospital of April 12 last, page 32, we gave an abbreviated report on the Hospital Saturday almoner system prepared by a special committee of the Hospital Saturday Fund. In another column we report the adjourned meeting of Delegates at which these recommendations were adopted by large majorities. In effect the decisions arrived at mean (1) that each patient when sent by the Hospital Saturday Fund to a Metropolitan hospital which employs an almoner must be accepted without inquiry on the production of a letter of recommendation from this Fund. Briefly the demand, then, is that each hospital shall suspend the almoner system in regard to all such cases. (2) That all Metropolitan hospitals which have been awarded grants by the Saturday Fund are requested to adopt this radical change of system and to admit all such patients without further inquiry. Failing compliance, the Distribution Committee is instructed to report to the Hospital Saturday Board the names of the hospitals who decline to agree to accept Hospital Saturday patients without passing through the almoner. On the receipt of the names of such hospitals it will be in order for the Saturday Fund Board to pass a resolution declining to award further grants to these institutions.

It will be evident to those most familiar with the history of the Hospital Saturday Fund and to all who have the interests of that Fund at heart that these decisions as they stand cannot be maintained and enforced, for they are not likely to promote the welfare of the Saturday Fund in the long run. It is most unreasonable that the delegates of the Saturday Fund should endeavour to force any hospital to alter its entire system of admitting patients at their will and pleasure. It is further an error of judgment not based upon knowledge for those who attended the recent meeting so to lose their heads as to pass a resolution which in effect threatens the hospitals—and they are the best administered hospitals in London which have adopted the almoner system—with the withdrawal of the support of the Hospital Saturday Fund unless they suspend that system in regard to all patients nominated by this Fund. If the Hospital Saturday Fund contributed 50 or 70 per cent. of the revenue of a particular hospital they might possibly venture on some such action towards the institution in question, but seeing that the actual sum distributed by the Metropolitan Hospital Saturday Fund amongst the whole of the Metropolitan hospitals amounted to less than 3½ per cent. of the ordinary income of these institutions, the unwisdom of the Board of Delegates in passing the third resolution becomes at once manifest. We, therefore, cordially hope that the best and most thoughtful minds on the Board of Delegates may be able to secure the adoption of wiser counsels in the near future. For the present the hospitals may be well content to manage their own affairs under the almoner system with the utmost care and consideration for all the interests concerned. Should an attempt be made to withdraw the contributions at present given to the great general hospitals throughout London such a policy can only have one end. The Hospital Saturday Fund, in such circumstances, so opposed will its policy be to the best interests of all who support and use our voluntary hospitals, must gradually disappear.

It is perfectly certain that no economic or other laws can justify this attempted coercion by the majority of the delegates of the Hospital Saturday Fund. The delegates appear to have been led away by the eloquence of a few men of some ability who failed to grasp from every point of view the subject they addressed themselves to, and who fell into the mistake of trying to compel compliance with their will and pleasure without any adequate apprehension of the grave dangers they were creating. It would be impossible for any hospital to consent to accept from anybody money which is hampered by conditions that would, in the opinion of the managers, seriously interfere with its proper administration. On more than one occasion great hospitals have refused considerable sums to which unacceptable conditions were attached. In the case of the Saturday Fund the amount of money contributed to the majority of Metropolitan hospitals is relatively so small as to be largely inappreciable. Add to this the fact that the Hospital Saturday Fund at present receives material value in the shape of tickets of admission, and the grievous error and injustice of the claims put forward by the Board of Delegates on the 17th instant must be apparent to every man of business, as well as to all who are familiar with the work and administration of the best hospitals.

